# Metamemory in a Familiar Place: The Effects of Environmental Context on Feeling of Knowing

**DOI:** 10.1037/xlm0000292

**Published:** 2016-06-09

**Authors:** Maciej Hanczakowski, Katarzyna Zawadzka, Harriet Collie, Bill Macken

**Affiliations:** 1 School of Psychology, Cardiff University

**Keywords:** feeling of knowing, context, metamemory

## Abstract

Feeling-of-knowing (FOK) judgments are judgments of future recognizability of currently inaccessible information. They are known to depend both on the access to partial information about a target of retrieval and on the familiarity of the cue that is used as a memory probe. In the present study we assessed whether FOK judgments could also be shaped by incidental environmental context in which these judgments are made. To this end, we investigated 2 phenomena previously documented in studies on recognition memory—a context familiarity effect and a context reinstatement effect—in the procedure used to investigate FOK judgments. In 2 experiments, we found that FOK judgments increase in the presence of a familiar environmental context. The results of both experiments further revealed still higher FOK judgments when made in the presence of environmental context matching the encoding context of both cue and its associated target. The effect of context familiarity on FOK judgment was paralleled by an effect on the latencies of an unsuccessful memory search, but the effect of context reinstatement was not. Importantly, the elevated feeling of knowing in reinstated and familiar contexts was not accompanied by an increase in the accuracy of those judgments. Together, these results demonstrate that metacognitive processes are shaped by the overall volume of memory information accessed at retrieval, independently of whether this memory information is related to a cue, a target, or a context in which remembering takes place.

Acts of learning and remembering always take place within a context, many aspects of which may be irrelevant or incidental to the focal content of the episode. Whereas a major body of research has investigated the influence of such contextual factors on the memory processes of encoding and retrieval (e.g., Murnane, Phelps, & Malmberg, 1999; Smith & Handy, 2014; Smith & Manzano, 2010), considerably less has been devoted to understanding how context may influence other processes involved in an individual’s evaluation, monitoring and regulation of such acts of learning and remembering. Such processes—that is, *metamemory* or more generally *metacognitive* processes—play a key role in determining outcomes such as the amount of time people spend trying to learn or retrieve information, their confidence in the veracity of retrieved information and—the focus of our current investigation—their belief that information that currently eludes recollection may nonetheless be retrieved in the future.

Failed retrieval attempts such as these are a prevalent feature of our memory in everyday life. We often fail to remember people’s names, most of the answers to questions in TV quizzes, or authors of papers important for our current work. These failures of memory access do not necessarily mean, however, that the relevant information is not available in our memory. Often, the relevant information is there but cannot be accessed with cues currently at our disposal. Importantly, people can often discriminate between questions for which answers are known but are temporarily inaccessible and questions for which answers are not known (Leonesio & Nelson, 1990; Morson, Moulin, & Souchay, 2015). The research on metamemory aspects of remembering has repeatedly addressed the issue of the basis of people’s conviction that certain inaccessible information is stored in memory—that is, their *feeling of knowing*—revealing that both the feeling of familiarity elicited by a memory question (Metcalfe, Schwartz, & Joaquim, 1993; Schwartz & Metcalfe, 1992) and any partial, incomplete, and sometimes even incorrect information about a sought-after target that comes to mind in the process of retrieval (Koriat, 1993, 1995) are relevant in this respect. In the present study we investigated whether the feeling that inaccessible information is nevertheless available in memory is shaped also by factors that are different from both the memory question that is asked and the partial information associated with what is to be retrieved. Specifically, we investigated whether this feeling is shaped by the features of an incidental context that accompanies memory questions.

People’s conviction that an inaccessible answer is or is not available in memory has been studied in laboratories by eliciting feeling-of-knowing (FOK) judgments. In a typical procedure, participants study cue–target pairs of words, and they are subsequently given a cued-recall task. In this cued-recall task, whenever a participant fails to retrieve a target in response to a given cue, a question concerning the probability of subsequent recognition of this target is posed. It is assumed that judgments provided in response to such a question—FOK judgments—tap into participant’s conviction that the relevant target is available in memory even though it is not currently accessible. Importantly, studies on FOK judgments have revealed them to be related to various decisions participants make in a memory task, such as when to terminate memory search (Malmberg, 2008; Singer & Tiede, 2008) and whether it is worth restudying a particular target in a preparation for subsequent tests (Hanczakowski, Zawadzka, & Cockcroft-McKay, 2014). These findings indicate that processes associated with FOK judgments play an important role in effective regulation of the process of remembering.

Research on the basis of FOK judgments has focused to date on two factors that determine participants’ predictions of future recognition. First, increasing cue familiarity leads to elevated FOK judgments, even though by and large it does not affect the actual probability of subsequent target recognition. In a procedure used by Schwartz and Metcalfe (1992), the standard FOK paradigm was supplemented with a prefamiliarization phase in which some words subsequently used in cued recall and FOK phases as both targets and paired cues were included among fillers in a pleasantness judgment task. Whereas prefamiliarization of target words did not affect FOK judgments in a subsequent FOK procedure, these judgments were found to be higher in response to primed rather than unprimed cues. These results point to a cue familiarity heuristic by which familiarity of a question serves as an indicator that an answer to this question is known. Conclusions concerning cue familiarity as the basis of FOK are strengthened by findings from related paradigms. For example, Reder (1987), who originally developed the priming manipulation for the investigation of quick assessments of knowledge that precede the actual retrieval attempts, found that people are more likely to quickly infer that they know the answer to a memory question if elements of this question have been previously primed. Koriat and Levy-Sadot (2001) extended the basic logic of cue familiarity to the domain of semantic knowledge by manipulating familiarity of the referents of general knowledge questions. As in the case of episodic memory, participants were more likely to provide high FOK judgments when the terms used in a memory question were more familiar.

Second, access to partial information about a target that may occur during an unsuccessful attempt to retrieve this target also affects FOK judgments. Koriat (1993) asked participants to study strings of four or five letters and then asked them both to retrieve as many letters as they could and to provide an FOK judgment for each string. The main conclusions were that FOK judgments were strongly related to the number of letters participants provided for each string and also that this relation held independently of whether provided letters were correct or not. Other studies provide additional evidence for the target accessibility account by demonstrating that FOK judgments are related to retrieval of the valence of the currently inaccessible target word (Schacter & Worling, 1985; Thomas, Bulevich, & Dubois, 2011) or other semantic aspects of the target word such as its activity (Koriat, Levy-Sadot, Edry, & de Marcas, 2003). Also, episodic information associated with the target which when retrieved does not allow for answering a specific memory question posed by the experimenter nonetheless increases FOK judgments. Thus, when asked to provide an FOK judgment concerning future recognizability of one source dimension for a given item, these FOK judgments are higher if information concerning another, unrelated source dimension is successfully retrieved (Brewer, Marsh, Clark-Foos, & Meeks, 2010). As a direct consequence of this sensitivity of FOK judgments to noncriterial recollection of target features, FOK judgments are also increased when targets are associated at encoding with more rather than fewer individual features (Schwartz, Pillot, & Bacon, 2014).

Both the cue familiarity and accessibility accounts emphasize the role of focal features of the memory task—that is, cues and targets—as determinants of FOK judgments. However, a memory task setting is never limited to the question that is asked and the answer that is sought but rather is always immersed in what may appear as an incidental context in the form of a particular place, mood of the rememberer, or thoughts that accompany it. In memory research such incidental contexts are commonly operationalized as incidental backgrounds for focal stimuli, either in the form of photographs (e.g., Murnane, Phelps, & Malmberg, 1999; Reder et al., 2013) or short films (e.g., Smith & Manzano, 2010). Although participants are not instructed to remember or even pay attention to these incidental contexts, their presence at retrieval and their relationship to contexts present at encoding have been found to affect both responding in memory tests and the accuracy of provided responses (see Smith & Vela, 2001, for a review). The question addressed here is how such nonfocal and incidental aspects of the setting which accompany encoding and retrieval influence metamemory processes associated with FOK judgments.

Our recent studies give credence to the idea that metamemory judgments in general may be sensitive to the manipulations of context. Hanczakowski, Zawadzka, and Coote (2014; see also Hanczakowski, Zawadzka, & Macken, 2015) investigated the effects of context on retrospective confidence judgments in a recognition memory task. Participants were presented with faces paired with individual context photographs of various landscapes, animals, buildings, and so forth. In a subsequent two-alternative forced-choice recognition test, they were asked to discriminate between studied and novel faces as well as to provide assessments of confidence in the accuracy of their recognition decisions. Faces in a memory test were presented in three different conditions. In the reinstated context condition, test faces were accompanied by the same context photograph with which the target face was presented at study. In the re-paired context condition, test faces were accompanied by a familiar context photograph that was presented with a different face at study. In the novel context condition, test faces were accompanied by a novel context photograph that was not included in the study phase. Crucial comparisons in this design were informed by previous studies that used this particular design for investigating context effects (Hockley, 2008; Macken, 2002; Murnane et al., 1999). A comparison of novel and re-paired context conditions reveals the influence of context familiarity on confidence judgments, whereas the comparison of reinstated and re-paired context conditions keeps context familiarity levels unchanged and so can illuminate the role of retrieval of item–context associations in driving confidence judgments.

The results obtained by Hanczakowski, Zawadzka, and Coote (2014) suggest that both context familiarity and context reinstatement affect retrospective confidence judgments in a number of ways. First, confidence was greater when context was familiar (in the re-paired context condition) rather than novel, even though context familiarity did not in fact affect the accuracy of recognition decisions. Second, confidence judgments were also higher when context was reinstated rather than re-paired. This last effect was accompanied by higher accuracy of recognition decisions in the reinstated context condition but only when either encoding of item–context associations was particularly strong or a recognition test was altered to be particularly sensitive to such associations (although see Hanczakowski et al., 2015). Together, these results indicate that increased context familiarity and augmented retrieval of item–context associations both translate into the type of metamemory process associated with higher retrospective confidence judgments.

The question that we investigate in the present study is whether similar effects of context familiarity and context reinstatement would shape prospective FOK judgments. The two factors shaping retrospective confidence judgments in our previous investigations into context effects seem conceptually related to factors known to influence FOK judgments. Regarding context familiarity, it is plausible that its role in FOK judgments would be similar to the role of cue familiarity described by Schwartz and Metcalfe (1992; see also Koriat & Levy-Sadot, 2001; Metcalfe et al., 1993; Reder, 1987). In a recognition test, increasing context familiarity inflates retrospective confidence for recognition decisions. Importantly, it has also been shown that when cue–target pairs are studied, prefamiliarizing the cues leads to inflated retrospective confidence judgments in a subsequent recognition test for targets, as long as cues are embedded in recognition trials (Chua, Hannula, & Ranganath, 2012; Hanczakowski, Pasek, Zawadzka, & Mazzoni, 2013). Given the similar influence of cue and context familiarity on confidence judgments, one can reasonably predict that context familiarity would also influence the magnitude of FOK judgments. However, there is an important distinction to be made between the roles of cues and contexts in the FOK paradigm. Specifically, participants are explicitly instructed to use cues to attempt retrieval of associated targets and also to use them in order to predict future recognizability of inaccessible targets. As such, focal, deliberate processing of cues is an integral part of the task setting. By contrast, contexts present at retrieval are incidental to the task. It thus remains possible that by virtue of being incidental, context would not have a similar impact on the processes underlying FOK, in which case context familiarity should not lead to elevated FOK judgments.

Regarding context reinstatement, tentative predictions can be derived from general ideas outlined by Koriat (1993) in his accessibility model, according to which, in the process of memory search for a target, accessing any additional information stored in memory elevates FOK judgments. Reinstating encoding context at the time of the test is known to facilitate retrieval (Macken, 2002), which suggests that context reinstatement may also boost FOK judgments, just as it increases retrospective confidence for recognition decisions (Hanczakowski, Zawadzka, & Coote, 2014). However, it is important to consider different ways in which reinstated context may impact on what is retrieved. Context at encoding becomes associated with both cues and their respective targets. Reinstating the same context at retrieval may thus facilitate full memory access to targets, as has been occasionally reported (e.g., Smith, Handy, Angello, & Manzano, 2014), but also partial access to some features of these targets. It is well established that access to partial information about targets not only boosts FOK judgments but also increases their relative accuracy (Koriat, 1993; Thomas et al., 2011). Thus, if context reinstatement were to increase FOK judgments by facilitating partial retrieval of target information, it should also increase the correlation between those judgments and performance in the recognition task.

Alternatively, context reinstatement may facilitate retrieval of associations between a particular context and the cue which was studied in this context. Such an outcome would be akin to the role of context reinstatement in recognition rather than cued recall. It remains an open question whether retrieval of such cue-to-context associations would boost FOK judgments. To the extent that retrieval of information about a cue is functionally similar to retrieval of noncriterial information about targets (see Brewer et al., 2010; Hertzog, Fulton, Sinclair, & Dunlosky, 2014) it should also inflate FOK judgments. Crucially, the predictive accuracy of FOK judgments should not benefit from retrieval of additional information about cues, since that accuracy is based on target information. Thus, if context reinstatement increases FOK judgments without affecting their accuracy, such an effect would be attributed to the impact on FOK judgments of the retrieval of information about the cue, as opposed to the target.

We present two experiments that aimed at assessing the role of contextual factors in shaping FOK judgments. In Experiment 1, we adapted the paradigm used in the investigations of context effects in recognition memory (Murnane et al., 1999) so that it could be used for eliciting FOK judgments. We thus had three levels of study–test context relationship—reinstated, re-paired, and novel—to assess the role of context familiarity and context reinstatement in shaping prospective FOK judgments elicited after unsuccessful attempts to retrieve target words. In Experiment 2, we repeated the same design for assessing FOK judgments while adding another experimental condition—a prefamiliarized context condition—in which context familiarity was manipulated via a preexposure procedure previously used in the studies on FOK judgments (Schwartz & Metcalfe, 1992; see also Reder, 1987; Reder & Ritter, 1992).

## Experiment 1

In the present experiment, the procedure used to investigate context effects in recognition, using the reinstated, re-paired, and novel context conditions, was adapted for use in the recall–judgment–recognition paradigm used to investigate FOK judgments. Participants studied cue–target pairs of words presented against the background of individual context photographs. In a subsequent cued–recall test, participants were presented with cues and asked to retrieve associated targets and provide FOK judgments for all cues for which the recall attempt was unsuccessful (i.e., no response was provided). Cues were presented with the reinstated, re-paired, or novel context photographs. The main interest was in the magnitude of FOK judgments in these three conditions. We predicted that context familiarity would affect FOK judgments, leading to higher judgments in the re-paired than novel context condition. We further predicted that context reinstatement would also affect FOK judgments, leading to higher judgments in the reinstated than in the re-paired context condition.

The main focus of the present study is on the factors affecting the magnitude of FOK judgments. Nevertheless, apart from that issue, the paradigm for eliciting FOK judgments allowed us to investigate three other theoretical problems. First, the FOK procedure includes a cued-recall phase for which the cue is the same as that used in the subsequent FOK judgment phase. Thus, the current procedure allows for examining context effects in cued-recall performance. Of particular interest here is the context reinstatement effect, that is, the potential benefits for correct recall conferred by reinstating the exact encoding context at retrieval. A meta-analysis conducted by Smith and Vela (2001) suggested that a small context reinstatement effect may sometimes be detected in cued-recall performance, although this effect was not significant for the data pooled across various studies.

Second, the procedure for eliciting FOK judgments commonly includes a final recognition test, which allows for assessing the accuracy of participants’ FOK judgments, that is, whether their judgment about the success or otherwise of a subsequent retrieval attempt turns out to be correct. We included a recognition test phase in our procedure as well. We expected the context familiarity manipulation to leave the accuracy of FOK judgments unaffected as performing cued recall in the presence of familiar contexts should not yield any additional information diagnostic of future recognition (see also Schwartz & Metcalfe, 1992). The prediction was less clear for the manipulation of context reinstatement. If context reinstatement facilitates retrieval of partial target information, we would expect it to increase the accuracy of FOK judgments as access to some features of the target should be diagnostic of subsequent recognition. However, if context reinstatement facilitates retrieval of cue-to-context associations but not partial retrieval of the target, then the accuracy of FOK judgments should remain unaffected by this manipulation.

Finally, one feature of FOK judgments is that their magnitude is often related to the duration of memory search in a preceding cued-recall task. Specifically, people are more motivated to continue memory search under conditions that are related to higher FOK judgments. This is often assessed by looking at latencies for unsuccessful retrieval attempts, which are longer when FOK judgments are higher (e.g., Malmberg, 2008). We looked at these latencies in the present study, expecting that any effect of context on the magnitude of FOK judgments would be also reflected in longer latencies to terminate an unsuccessful memory search in the cued-recall test.

### Method

#### Participants

Forty-two undergraduates from Cardiff University participated in the present experiment.

#### Materials and design

A cohort of 120 words of medium frequency and concreteness was chosen from the MRC Psycholinguistic Database (Coltheart, 1981). They were randomly paired to create 60 cue–target pairs. An additional 300 words were chosen to serve as foils in a recognition test. A set of 80 black-and-white pictures of landscapes, buildings, and animals was assembled from various Internet sources to be used as context photographs. Sixty of these pictures were used as context photographs for cue–target pairs presented at study, while the remaining 20 were used in the novel context condition at test.

Three experimental conditions were created by varying context photographs in the cued-recall and FOK judgment phases of the procedure. An equal number of cues used to elicit target recall and FOK judgments were presented in each of (a) the reinstated context condition, in which context photograph was the same as presented for this cue in the study phase, (b) the re-paired context condition, in which context photograph was taken from a different cue–target pair, and (c) the novel context condition, in which an unstudied context was presented. The context conditions were manipulated within participants and the assignment of word pairs to conditions was counterbalanced across participants.

#### Procedure

The experiment consisted of three phases. In the study phase, participants were presented with cue–target word pairs for study. Each word pair was presented for 2.5 s with a 500-ms interstimulus interval. Words were presented in red font, superimposed on black-and-white photographs, with cues presented at the top and targets and the bottom of the photograph. Participants were instructed to learn pairs of words for a future memory test. The study phase was immediately followed by a cued-recall test in which cues were presented against the background of context photographs (at the top of the picture). Participants were asked to recall targets associated with cues by typing them via the computer keyboard and to type in the word *blank* if they were unable to recall the target word. If the word *blank* was typed in, the cue and its context photograph were presented again and the participant was asked to judge the probability (on a scale of 0%–100%) that the target would be recognized in a subsequent recognition test. The time to respond in cued-recall and judgment steps of the test was not limited. The cued-recall test was immediately followed by a recognition test. In each trial of a recognition test, one of the targets was presented along with five distracter words (a six-alternative forced-choice test, 6AFC). Cues and context photographs were not presented at recognition, and the time to respond in this test was not limited.

### Results and Discussion

Descriptive statistics are presented in Table 1.[Table-anchor tbl1]

#### Cued recall

Performance in the cued-recall phase was analyzed with a one-way analysis of variance (ANOVA) with the proportion of correctly recalled targets as a dependent measure. This analysis revealed a main effect of context condition, *F*(2, 82) = 33.12, *MSE* = .008, *p* < .001, η_p_^2^ = .45. Given that we had no clear predictions concerning context effects in cued recall, we performed follow-up multiple comparisons with a Bonferroni-corrected level of α = .017. Cued-recall performance was best in the reinstated context condition, which was significantly better than both the novel context condition, *t*(41) = 4.29, *SE* = .02, *p* < .001, *d* = 0.68, and the re-paired context condition, *t*(41) = 7.96, *SE* = .02, *p* < .001, *d* = 1.24. Perhaps more surprisingly, correct cued-recall performance was also lower in the re-paired than in the novel context condition, *t*(41) = 3.91, *SE* = .02, *p* < .001, *d* = 0.61, suggesting that presenting re-paired contexts at test interfered with retrieval of targets associated with the cues. Indeed, a one-way ANOVA conducted on the rate of intrusions in cued recall revealed differences between conditions, *F*(1, 82) = 19.56, *MSE* = .01, *p* < .001, η_p_^2^ = .32. Specifically, the rate of intrusions was higher in the re-paired context condition than in the both novel, *t*(41) = 5.70, *SE* = .02, *p* < .001, *d* = 0.88 and reinstated context conditions, *t*(41) = 4.49, *SE* = .02, *p* < .001, *d* = 0.73. The rate of intrusions did not differ between novel and reinstated context conditions (*t* < 1.3, *p* > .22). The fact that interference was observed in the re-paired context condition should be remembered when interpreting the results for FOK judgments, as we discuss later.

#### FOK judgments

The results of main interest concern the magnitude of FOK judgments. The means of FOK judgments made after unsuccessful retrieval attempts were analyzed with a one-way ANOVA, which revealed a significant main effect of context condition, *F*(2, 82) = 26.52, *MSE* = 27.56, *p* < .001, η_p_^2^ = .39. Planned comparisons revealed that FOK judgments were higher in the re-paired than in the novel context condition, *t*(41) = 5.80, *SE* = 0.86, *p* < .001, *d* = 0.87, as well as being higher in the reinstated than in the re-paired context condition, *t*(41) = 2.66, *SE* = 1.25, *p* = .011, *d* = 0.41. These two results are in line with our initial predictions that both context familiarity and context reinstatement would increase FOK judgments.

To assess whether these effects on metacognitive monitoring were also manifested in a measure of metacognitive control, we examined the time to end an unsuccessful memory search—that is, the latencies for *blank* responses to recall cues—in all context conditions. A one-way ANOVA revealed a significant effect of context condition on these latencies, *F*(2, 82) = 4.03, *MSE* = 1.33, *p* = .021, η_p_^2^ = .09. Planned comparisons assessed latencies as a function of context familiarity and context reinstatement. A comparison between re-paired and novel context condition was not significant, *t*(41) = 1.91, *SE* = .25, *p* = .06, *d* = 0.31, but a numerical trend (which was significant in Experiment 2) suggested longer termination latencies for the re-paired context condition. This effect was expected inasmuch as it parallels the predicted effect that context familiarity had on the magnitude of FOK judgments. However, despite the effect of context reinstatement on FOK judgments, there was no difference between re-paired and reinstated context conditions in the duration of unsuccessful retrieval attempts (*t* < 1).

We also assessed participants’ ability to predict future recognition by examining gamma correlations between FOK judgments for unrecalled items and subsequent recognition performance for the same items. Generally, gammas were low and in fact none of them was significantly different from zero (*p* = .13, *p* = .99, and *p* = .64, for the reinstated, re-paired, and novel context conditions, respectively). Although previous research has generally documented positive gammas, indicating that participants are able to predict accurately future recognition (e.g., Sacher, Landré, & Taconnat, 2015; Souchay, Moulin, Clarys, Taconnat, & Isingrini, 2007; Thomas, Bulevich, & Dubois, 2012), it is also worth noting that gammas for newly learned episodic information are generally on the low side, at least in comparison to those found with FOKs for semantic information (Nelson & Narens, 1990; Schwartz & Metcalfe, 1992). Also, the procedure of collecting FOK judgments only for unrecalled items, although most commonly used in the FOK literature, is bound to result in underestimation of people’s ability to predict subsequent recognition (cf. Koriat, 1993; Reder, 1987). A one-way ANOVA failed to reveal any differences in gammas among the three context conditions (*F* < 1).^1^ Therefore, not only did context not affect how predictive FOKs were of subsequent retrieval in Experiment 1, but FOKs were generally inaccurate throughout.

#### Recognition

As our primary interest in the present study lies in the processes occurring when retrieval attempts fail, we restricted the analysis of recognition performance to items for which participants responded *blank* in the cued-recall phase (and thus for which FOK judgments were collected). While the recognition test itself did not contain any context picture, a one-way ANOVA on recognition accuracy for these items revealed a significant main effect of the study/recall test context condition, *F*(2, 82) = 7.41, *MSE* = .02, *p* = .001, η_p_^2^ = .15. Bonferroni-corrected multiple comparisons (α = .017) revealed that recognition performance for items that previously served as targets in the re-paired context condition was better than performance for targets previously assigned to the reinstated context condition, *t*(41) = 3.82, *SE* = .03, *p* < .001, *d* = 0.55. It also tended to be better than performance for targets previously serving in the novel context condition, but this difference was not statistically significant, *t*(41) = 2.29, *SE* = .03, *p* = .03, *d* = 0.35. The latter two conditions did not differ from each other (*t* < 1.7, *p* > .10). These recognition results are consistent with the idea that interference was present in cued recall for the re-paired context condition, increasing the chances that no response would be produced on some trials for this condition even when a target was actually encoded in memory and could be endorsed in a recognition test.

The main result of the present experiment is that FOK judgments are affected by the incidental context in which they are made. FOK judgments were higher in the presence of a familiar context in the re-paired context condition than in the presence of an unfamiliar context in the novel context condition. This particular effect was reflected also in the measure of metacognitive control as participants searched for a target for longer in the presence of a familiar context. FOK judgments were further increased when the original study context was reinstated, although in this case no effect on the measure of metacognitive control was observed. The results obtained here for FOK judgments parallel those observed for retrospective confidence judgments in the study by Hanczakowski, Zawadzka, and Coote (2014; see also Hanczakowski et al., 2015), revealing that metacognitive measures are consistently sensitive to context effects. Therefore, context influences a range of processes taking place during remembering and learning in both retrospective evaluation of retrieved information and prospective judgments about the memorability of currently inaccessible information, as well as influencing the amount of time that participants spend trying to retrieve information.

One unexpected finding in Experiment 1 concerns memory performance in the re-paired context condition. In recognition studies, a comparison of this condition to a novel context condition commonly fails to reveal any differences (Hockley, 2008; Hockley, Bancroft, & Bryant, 2012; Macken, 2002), and one recent study actually revealed better recognition performance in the re-paired context condition (Bloch & Vakil, 2016). Against this background it is somewhat surprising that cued-recall performance in the present experiment showed reduced performance in the re-paired compared to the novel context condition. However, a plausible explanation for this is that including a re-paired context along with the cue word in a cued-recall test may lead to activation of the target originally paired with that context, increasing memory interference and thus lowering performance. The analysis of intrusion rates—which were higher in the re-paired than the other context conditions—and subsequent recognition are consistent with this interference account.

The crucial point about the putative interference observed in our results is that it clouds the interpretation of the FOK pattern in this condition. This is particularly important for the evaluation of the context familiarity hypothesis. We have argued that a difference in FOK judgments between re-paired and novel context conditions would reveal the role of context familiarity in metacognitive monitoring. However, additional interference occurring in the re-paired context condition means that such a difference is also predicted by the accessibility account of FOK judgments (Koriat, 1993). This model explicitly assumes that any information retrieved from memory, be it correct or incorrect, raises FOK judgments. If retrieval interference is the reason for the lower cued-recall performance in the re-paired context condition, then not only is it likely to lead to more intrusions, but it may also point to the retrieval of more partial information, albeit partial information about the incorrect target previously associated with the re-paired context. This in turn could lead to an increase in FOK judgments, rather than them being increased directly by the familiar context itself. In light of these results, an appropriate test to distinguish between an effect due to context familiarity and one due to an increase in partial retrieval requires a condition in which context is familiar but not associated with any target from encoding, and therefore cannot serve as a cue to the partial retrieval of any study items. In Experiment 2 we included such a condition, utilizing the context preexposure procedure similar to that which has been previously used to investigate the role of cue familiarity in shaping FOK judgments (Hanczakowski et al., 2013; Schwartz & Metcalfe, 1992).

## Experiment 2

The aim of the present experiment was threefold. First, we wanted to replicate the main findings concerning the effects of context on FOK judgments from Experiment 1, and thus we included the same three context conditions—reinstated, re-paired, and novel—in the present design. We again expected to document higher FOK judgments in the re-paired than in the novel context condition and still higher FOK judgments in the reinstated context condition. A more exploratory aim was to assess whether we would replicate the context reinstatement and interference effects in cued-recall performance.

The second aim of the experiment was to provide an additional test of the context familiarity hypothesis. To this end, we included a fourth condition in the experimental design—a prefamiliarized context condition—in which contexts were made familiar via a preexposure (priming) procedure (Reder, 1987; Schwartz & Metcalfe, 1992). Therefore, the study phase was preceded by a context familiarization phase in which participants were presented with black-and-white photographs for which they were asked to provide pleasantness judgments. No words were superimposed on any of the photographs in this phase. A subset of these photographs was later used as context photographs in the cued-recall and FOK judgment phases of the experimental procedure. We assumed that these contexts would be familiar due to their exposure in the familiarization phase, but given that they were never associated with any target, there should be no additional partial retrieval of any target for these contexts. The question of interest is whether FOK judgments given in the presence of these prefamiliarized contexts would be higher than FOK judgments given in the presence of novel contexts, as the context familiarity hypothesis would predict.

The third aim of the experiment was to provide additional insight into the relative accuracy of FOK judgments in various context conditions. In Experiment 1 we found that although varying context at retrieval affected the magnitude of FOK judgments, it did not have any effect on how accurately these judgments predicted subsequent recognition. The interpretation of this null result is, however, clouded by the floor effect given the fact that participants’ ability to predict subsequent recognition of unrecalled targets was no different than chance in all conditions. In the present experiment, we modified the recognition testing procedure in order to increase the relative accuracy of metacognitive judgments. Whereas in Experiment 1 only target words were presented on the 6AFC test, and participants’ task was only to recognize a target on each test trial, in Experiment 2 both cue and target were represented in the recognition test accompanied by the same context with which they appeared in the cued-recall/FOK phase. We reasoned that participants’ ability to predict their recognition performance should be better when predictions are made under more similar conditions to those that are later used in the recognition test. Of interest is whether context manipulations would affect the relative accuracy of FOK judgments under these conditions.

### Method

#### Participants

Fifty-two undergraduates participated in the present experiment.

#### Materials and design

The materials were the same as in Experiment 1, except that a novel set of 35 black-and-white context photographs was assembled from various Internet sources. Four conditions were used in the present study, which meant that 15 items, rather than 20 used in Experiment 1, were assigned to each experimental condition. Five of the context photographs used in the novel context condition of Experiment 1 were added to the new set of 35 photographs, and were used for the preexposure procedure in the present experiment. A random set of 15 of these 40 prefamiliarized photographs was used in cued-recall, FOK judgment, and recognition phases to create the prefamiliarized context condition. The prefamiliarized context condition was included along with the reinstated, re-paired, and novel context conditions, which were the same as in Experiment 1. The assignment of word pairs to these four experimental conditions was counterbalanced across participants.

#### Procedure

The procedure was the same as in Experiment 1, except for the addition of the preexposure phase and the changes in the recognition test. In the preexposure phase, participants were presented with individual black-and-white photographs, and they were asked to judge the pleasantness of each photograph on a scale of 1–5. The time to make a pleasantness judgment was not limited. On each trial of a recognition test, participants were presented with one of the targets together with five distracter words. Additionally, in the present experiment a cue for each of the targets was presented in each recognition trial. This cue was superimposed on the same context (reinstated, re-paired, novel, or prefamiliarized) with which it was presented in the preceding cued-recall and FOK judgment phases of the experiment.

### Results and Discussion

Descriptive statistics are presented in Table 1.

#### Cued recall

A one-way ANOVA revealed a main effect of context condition on cued-recall performance, *F*(3, 153) = 2.72, *MSE* = .01, *p* = .046, η_p_^2^ = .05. Given a large number of possible comparisons with four experimental conditions, we focused here only on the conditions included also in Experiment 1 to assess the replicability of the context reinstatement and interference effects. We excluded the prefamiliarized condition, as it was irrelevant for this analysis. The Bonferroni-corrected comparisons of reinstated, re-paired, and novel context conditions (α = .017) revealed only that correct recall was higher in the reinstated than in the novel context condition, *t*(51) = 2.61, *SE* = .02, *p* = .012, *d* = 0.35. This result suggests that reinstating context yields benefits for cued-recall performance, but it should be interpreted with caution, as a comparison between reinstated and re-paired context conditions, which keeps context familiarity equal and varies only cue-to-context match, was not significant, *t*(51) = 1.67, *SE* = .02, *p* = .10, *d* = 0.22, even though it was numerically in the direction favoring performance in the reinstated context condition. Interestingly, the difference between the re-paired and novel conditions was not significant (*t* < 1.3, *p* > .20), and performance in this experiment was numerically higher in the re-paired context condition. This comparison indicates that an unexpected effect of interference in the re-paired context condition that we observed in Experiment 1 was not replicated here. The lack of interference was also visible in the analysis of intrusions, which in contrast to Experiment 1 failed to reveal any difference between context conditions employed in the present experiment (*F* < 1).

#### FOK judgments

The magnitude of FOK judgments given after unsuccessful retrieval attempts was also analyzed with a one-way ANOVA, which revealed a main effect of context condition, *F*(3, 153) = 13.01, *MSE* = 59.98, *p* < .001, η_p_^2^ = .20. We first conducted planned comparisons to assess if the effects observed in Experiment 1 were replicated here. These comparisons revealed that FOK judgments were indeed higher in the re-paired than in the novel context condition, *t*(51) = 2.63, *SE* = 1.44, *p* = .011, *d* = 0.36, and also that FOK judgments were higher in the reinstated than in the re-paired context condition, *t*(51) = 2.39, *SE* = 1.46, *p* = .021, *d* = 0.33. These two results replicate Experiment 1. Of interest was also whether increasing context familiarity via preexposure would increase FOK judgments. A comparison of FOK judgments in the prefamiliarized context condition against FOK judgments made in the presence of unfamiliar context in the novel context condition revealed that this was indeed the case, *t*(51) = 5.23, *SE* = 1.66, *p* < .001, *d* = 0.73.

We again assessed whether these effects on metacognitive monitoring were also reflected in a measure of metacognitive control in the form of latencies to terminate an unsuccessful memory search. A one-way ANOVA revealed a main effect of context condition on latencies to respond *blank*, *F*(3, 153) = 3.50, *MSE* = 1.25, *p* = .017, η_p_^2^ = .06. In Experiment 1, we found that latencies reflected the effect of context familiarity but not context reinstatement. The same was the case in the present experiment, as latencies were shorter in the novel context condition compared both to latencies in the re-paired context condition, *t*(51) = 2.22, *SE* = 0.21, *p* = .031, *d* = 0.32, and latencies in the prefamiliarized context condition, *t*(51) = 3.36, *SE* = 0.18, *p* = .001, *d* = 0.48. At the same time latencies were not significantly different between reinstated and re-paired context conditions (*t* < 1).

We also assessed participants’ ability to predict future recognition by examining gamma correlations between FOK judgments to unrecalled items and subsequent recognition performance for the same items. Generally, gammas were higher in the present experiment than they were in Experiment 1, indicating that presenting retrieval cues in the same form when making an FOK judgment as is presented when performing the criterial test promotes the accuracy of metacognitive monitoring. In the present experiment gammas were positive and significantly different from zero in three out of four experimental conditions, the only exception being the novel context condition (*p* = .023, *p* < .001, *p* = .099, *p* = .015, for the reinstated, re-paired, novel, and prefamiliarized context conditions, respectively). The comparisons of gammas across context conditions failed to reveal any significant differences (*F* < 1).^2^

#### Recognition

We again restricted the analysis of recognition performance to items for which no response was produced in the cued-recall phase, and thus for which FOK judgments were collected. A one-way ANOVA on recognition accuracy for these items failed to reveal any significant differences between context conditions (*F* < 1). This result contrasts with the results of Experiment 1, in which recognition performance was highest for the re-paired context condition. The lack of this effect in the present experiment is consistent with the cued-recall data inasmuch as it again suggests that there was no interference in the re-paired context condition under conditions of the present experiment.

Experiment 2 replicated the crucial results from Experiment 1 concerning FOK judgments which were once again elevated both by context familiarity and context reinstatement. Additional support for the role of context familiarity in driving FOK judgments came from the prefamiliarized context condition. In this condition, context familiarity was increased via the preexposure manipulation and yet the results for FOK judgments paralleled those for the re-paired context condition in which context familiarity was increased via presentation with another target at study: FOK judgments for both of these conditions were higher compared to FOK judgments given in the presence of an unfamiliar context in the novel context condition.^3^ Importantly, also replicating Experiment 1, the effect of context familiarity was reflected in the measure of metacognitive control—the duration of memory search—although the effect of context reinstatement was not. Together, these results demonstrate once more the sensitivity of metacognitive monitoring and control to the environmental context in which these metacognitive processes unfold.

The results of Experiment 2 demonstrated also once more that although context shapes the magnitude of FOK judgments, it does not affect their relative accuracy. In the present experiment, as opposed to Experiment 1, a match in cuing conditions was preserved between FOK judgment and recognition phases of the experimental procedure. This match produced a clear positive relationship between FOK judgments and later recognition performance, one that was absent in Experiment 1. Still, the relationship between the contexts present at retrieval and at encoding did not affect the relative accuracy of FOK judgments.

One effect that was clearly not replicated in the Experiment 2 was the apparent interference effect in the re-paired context condition. In Experiment 1, it was reflected in the fact that the re-paired context condition was characterized by the lowest correct cued recall, the highest rate of cued-recall intrusions, and the highest rate of recognition for previously unrecalled items. All of these effects were absent in Experiment 2. Although this lack of interference in the re-paired context condition clearly facilitates the interpretation of one the central results of the present investigation, namely the role of context familiarity in driving FOK judgments, it remains unclear why interference was present in Experiment 1 but not in Experiment 2. In the General Discussion we return to these unexpected findings.

## General Discussion

The present study looked at the effects of context on FOK judgments. Based on our previous research, in which sensitivity of retrospective confidence judgments to context effects was revealed (Hanczakowski, Zawadzka, & Coote, 2014; Hanczakowski et al., 2015), we predicted that metacognitive monitoring of unsuccessful retrieval attempts may be likewise influenced by local context in which this monitoring takes place. The results confirmed our predictions, revealing two distinct effects that context exerts on FOK judgments. First, FOK judgments were influenced by context familiarity, as participants consistently offered higher predictions of later recognition of unrecalled targets in the presence of a familiar rather than novel context. This occurred both when context familiarity accrued during the study phase (a comparison of the re-paired and novel context conditions in Experiments 1 and 2) and when it accrued in a separate experimental phase in which some contexts were preexposed (a comparison of the prefamiliarized and novel context conditions in Experiment 2). Second, FOK judgments were influenced by context reinstatement as participants consistently offered higher predictions of later recognition of unrecalled items when a cue was coupled with the same context as at encoding rather than a familiar but switched context.

The main purpose of the present study was to elucidate the basis of FOK judgments. The first theories of FOK judgments postulated a specialized mechanism that provides direct access to the contents of a memory store and which would thus allow for accurate predictions of subsequent recognition (Hart, 1965). Such a direct-access theory predicts that the same factors that affect memory as assessed by cued recall or recognition would exert parallel influence on FOK judgments. Numerous findings from the literature on FOK judgments refute this prediction (e.g., Metcalfe et al., 1993) and the results of the present study join them in doing so by revealing that context dissociates the measures of FOK and memory. More recent theories of FOK judgments stress that people use a variety of cues present during the retrieval process to assess whether to-be-remembered items are actually stored in memory (Koriat, 1993; Schwartz & Metcalfe, 1992). The present study demonstrates that incidental contexts present at retrieval might provide some of these cues. By revealing the context familiarity and context reinstatement effects, the study extends the known basis of FOK judgments.

Starting with the context familiarity effect, the present study builds upon previous studies documenting the role of cue familiarity for FOK judgments (e.g., Metcalfe et al., 1993; Koriat & Levy-Sadot, 2001; Reder, 1987; Reder & Ritter, 1992; Schwartz & Metcalfe, 1992) but extends them by showing that nonfocal, incidental aspects of the setting may also enter in the processes giving rise to metacognitive judgments. The role of cue familiarity in shaping FOK judgments is perhaps less than surprising as one can plausibly infer that familiarity with a question should be related to familiarity with an answer to this question. After all, cues and their targets are always studied at the same time, and thus one could legitimately assume that good memory for a cue indicates effective encoding of an entire pair. The legitimacy of this assumption can be gleaned from studies on source memory in which retrieval of various aspects of a single study word (e.g., gender of a speaker and position on the screen) are stochastically dependent, showing improved performance for one aspect of the word with successful retrieval of the other aspect (e.g., Hicks & Starns, 2016). Such stochastic dependence is easily accounted for by fluctuations of attention during study episode, which also predicts a correlation in memory for cues and their targets. In the FOK procedure including cue priming (e.g., Schwartz & Metcalfe, 1992) participants are effectively misled as increased cue familiarity no longer reflects successful encoding at study and thus it does not need to relate to increased probability of successful encoding of a target associated with this particular cue.

By contrast, good memory for context is a generally much less obvious indicator of good memory for a target tested in this context. Context is by definition incidental to the tested target and thus, in contrast to a cue, may or may not be related to the context that was present at encoding of this target. Indeed, in our Experiment 1, only a third of retrieval contexts matched encoding contexts and this ratio was lowered to a quarter in Experiment 2. If targets were not studied in particular retrieval contexts, as in all context conditions except for the reinstated context condition, then obviously familiarity of these contexts has no bearing on how well a to-be-remembered target was encoded. A question thus arises why participants are still willing to base their FOK judgments on context familiarity. One possibility is that they do not realize that not every context is reinstated from the study phase. This, however, seems unlikely given the ratios mentioned earlier and also the presence of novel contexts which should generally alert participants to changes in contexts between encoding and retrieval. A more likely possibility relates to an alternative approach to the role of cue familiarity developed by Reder and her colleagues (Reder, 1987; Reder & Ritter, 1992; Reder & Schunn, 1996), by which reliance on cue familiarity is not a deliberate strategy but a simple and fast heuristic that needs not to be conscious. If people are generally prone to automatically assume that greater familiarity of a question is associated with better memory for an answer, then they may not parse a global sense of familiarity as originating from the focal (cue) and incidental (context) aspects of a retrieval setting. In this scenario, context familiarity is effectively misattributed to the question embedded in this context, giving rise to an effect that is indistinguishable from the effect of increased familiarity of the cue itself. Context familiarity effects for FOK judgments would thus be similar to effects obtained in recognition memory in which recognition probes presented in familiar context seem more familiar themselves, an effect revealed by an increase in both hits and false alarms in the presence of familiar contexts (Hockley, 2008; Murnane & Phelps, 1993).

The second effect of context on FOK judgments was revealed by looking at context reinstatement. Not only were FOK judgments higher in the presence of familiar compared to unfamiliar contexts, but they were still higher if these familiar contexts were the same contexts that were present at encoding for given pairs. Just as the effect of context familiarity on FOK judgments parallels a similar effect observed for retrospective confidence judgments in recognition decisions, so does the effect of context reinstatement (Hanczakowski, Zawadzka, & Coote, 2014). In the recognition studies, reinstating context facilitates retrieval of associations between a recognition target and its context and such retrievals constitute evidence that a given recognition item was studied. However, in the cued-recall test which was used in the present study, there are two words associated with each context: a cue and a target. Reinstating context can thus facilitate retrieval of an association between a cue and its initial context, just as in recognition studies. Alternatively (or additionally), it may facilitate retrieval of a to-be-remembered target itself. This latter possibility finds support from the cued-recall results that are discussed later. For the present point it is crucial to note that access to the target need not be complete and context reinstatement may also facilitate access to partial information about the target, which is known to affect FOK judgments (Koriat, 1993). Context reinstatement can thus have two different memory effects in a cued-recall situation and it is thus of interest which one is responsible for an increase in FOK judgments observed in the present study. The pattern of relative accuracy of FOK judgments is helpful in this regard. Previous research by Thomas et al. (2011) has consistently shown that partial retrieval of target information affects not only the magnitude of FOK judgments but also their relative accuracy. Specifically, retrieval of partial information is related to increased accuracy of FOK judgments. However, in the present study we failed to find any effects of context condition on the accuracy of FOK judgments. This strongly suggests that context reinstatement does not lead to retrieval of any information that would aid predictions regarding the outcome of subsequent target recognition. We thus argue that what participants retrieve in the reinstated context condition—and what is responsible for the elevated FOK judgments—is not partial information about the target but rather additional information about the cue, namely its association with the local context.

The important insight from our context reinstatement manipulation is thus that reinstating context at retrieval can facilitate access to various elements of the encoding episode, affecting metamemory assessments in turn. Recognition studies, on which the majority of recent work on context effects has concentrated (see Gruppuso, Lindsay, & Masson, 2007; Hockley, 2008; Levy, Rabinyan, & Vakil, 2008; Macken, 2002; Reder et al., 2013; Tibon, Vakil, Goldstein, & Levy, 2012), provide a very simplified scenario in which only a single focal memory item (the recognition probe) is presented and thus the role of context can only be determined as it applies to this one item. In cued recall—for which studies to date considered only the role of context in accessing to-be-remembered target (e.g., Smith et al., 2014)—context reinstatement can affect both how effectively the target is accessed but also how the cue is processed, as revealed in our present study. In more complex tasks, just as in rich environments outside laboratories, context effects may be more complex still. Our recent investigation of context reinstatement effects in an eyewitness scenario (Krogulska, Skóra, Scoboria, Hanczakowski, & Zawadzka, 2016) revealed that context reinstatement can reduce “do-not-know” responding for unanswerable questions for which correct answer was never presented to participants. Clearly, reinstating context could not facilitate retrieval of any information related to a detail that was never presented. However, reinstated context could facilitate retrieval to other aspects of stimuli rich in detail that are used in eyewitness settings, and those additional retrievals could influence participants’ metamemory assessments made for unanswerable questions. These context effects bear resemblance to noncriterial recollection effects observed for FOK judgments (Brewer et al., 2010; Hertzog et al., 2014; Schwartz et al., 2014) and extend them by demonstrating that metamemory processes aimed at one element of an episode need not only be affected by retrieval of information related to this particular element but can indeed be affected by noncriterial retrieval of any element of the particular episode.

Our results indicate that metacognitive judgments are sensitive to any type of memory information available at retrieval, whether elicited by cue, target, or context. Importantly, this sensitivity is not necessarily accompanied by changes in the accuracy or efficiency with which subsequent actual memory responses are made. The first aspect of this claim is generally in accordance with the ideas outlined in the accessibility framework developed by Koriat (1993; see also Koriat, 1995; Koriat & Levy-Sadot, 2001). In Koriat’s (1993) words,

Whenever subjects interrogate their memory for a specific piece of information, a variety of clues come to mind. These include activations from the terms in the question; structural, contextual, and semantic attributes; fragments of the target; and so on . . . even when retrieval fails, these very clues contain important information that can be used in judging whether the target can be recalled or recognized in the future. (p. 611)

However, what our results add to this observation is that when people judge the probability of future memory success, they seem to rely also on cues that do not contain any predictive information whatsoever, be it familiarity of cue and context or retrieval of information which is unrelated to retrieval of a target. As a result, the factors influencing metamemory judgments are not necessarily positively correlated with factors affecting successful recognition of target information. Instead, we propose that people do not actually evaluate the usefulness of any memory information that is accessed, but rather follow a very simple accessibility heuristic by which any type of memory information that they retrieve induces them to predict better memory performance in the future. We also note that the same occurs for postdictions of memory performance since context familiarity affects retrospective confidence judgments even when it has no impact on memory accuracy (Hanczakowski, Zawadzka, & Coote, 2014). Recently, Starns, Pazzaglia, Rotello, Hautus, and Macmillan (2013; see also Starns & Ksander, 2016) have shown that stronger memory for an item itself increases participants’ confidence in source decisions concerning the item, even though better memory that an item was presented clearly does not help answering the question about, for example, the voice in which it was presented. Together, all these results show sensitivity of metacognitive monitoring to any type of memory information elicited while making a metacognitive judgment.

In the metacognitive framework of remembering it is always assumed that metacognitive monitoring is important because it feeds into control processes that eventually impact upon various measures of memory performance. For example, the investigation of retrospective confidence judgments is vital as confidence assessments determine the likelihood that certain information will be included in a memory report (Hanczakowski et al., 2013; Koriat & Goldsmith, 1996). FOK judgments are known to be related to at least two forms of metacognitive control: the duration of a memory search (Singer & Tiede, 2008) and the propensity to choose unrecalled items for restudy (Hanczakowski, Zawadzka, & Cockcroft-McKay, 2014). Our variant of a “total accessibility” hypothesis outlined above would suggest that eliciting any type of memory information at retrieval (and possibly also at encoding) would be reflected in changes in how metacognitive control is exerted. This was clearly the case in our previous investigations of context effects in recognition in which both context familiarity and context reinstatement not only increased retrospective confidence judgments but also translated into more responses volunteered in a memory report. In the present study we looked at the latencies of an unsuccessful memory search in various context conditions. We found that context familiarity consistently affected metacognitive control as participants were motivated to search their memory longer in the presence of a familiar rather than novel context. Surprisingly, context reinstatement did not have any additional impact on metacognitive control beyond that already attributable to context familiarity. Even a combined analysis of search latencies for unsuccessful retrieval attempts in Experiments 1 and 2 with a 2 (context: reinstated vs. re-paired) × 2 (Experiment: 1 vs. 2) mixed ANOVA failed to reveal any effect of context reinstatement, *F*(1, 92) = 1.08, *MSE* = 1.58, *p* = .30, η_p_^2^ = .01. It is not clear at present why one form of metacognitive control is differently sensitive to two manipulations that have clearly similar effects on metacognitive monitoring. This issue will require further research but at present our findings clearly underscore the necessity of combining examinations of metacognitive monitoring with those of metacognitive control, as these constructs may be not always be equally sensitive to various experimental manipulations.

The present study was concerned with effects of context on metacognition but, as argued throughout this discussion, these are closely interweaved with context effects on memory, to which we now briefly turn. The paradigm we used in the present study was originally developed to examine context effects in recognition memory (Hockley, 2008; Macken, 2002; Murnane et al., 1999). In these studies, it is usually found that varying context familiarity exerts a similar effect on hit rates and false alarm rates, leaving recognition discrimination unchanged. An additional effect of context reinstatement is sometimes found, such that recognition discrimination is better in the presence of a reinstated rather than re-paired context, but this effect seems to be weak and is most often obtained when encoding instructions require participants to associate items with their contexts (e.g., Gruppuso et al., 2007; Hockley, 2008; Reder et al., 2013). The results of the present study can be discussed in relation to the effects of context reinstatement and context familiarity in cued recall. Regarding context reinstatement, the conclusion seems to be rather clear as in both experiments cued-recall performance was numerically highest in the reinstated context condition (differing significantly from the novel context condition in both Experiments 1 and 2), consistent with the idea that context reinstatement facilitated retrieval of targets (see also Smith et al., 2014). Interestingly, this occurred despite the fact that in none of the experiments participants were asked to associate word pairs with context photographs. These results confirm that even incidentally encoded context may benefit memory performance if reinstated at test.

The effects of context familiarity on cued recall in our experiments are more difficult to explain. The problem lies in the fact that these were inconsistent across experiments. In Experiment 1, we unexpectedly obtained results suggesting that re-paired context increased retrieval interference. Participants’ correct cued recall was the poorest in the re-paired condition while rate of intrusions was the highest. Subsequent recognition for unrecalled items revealed the highest performance in the re-paired context condition, consistent with the idea that some of targets that were encoded and thus available in memory were nevertheless not accessed in a cued-recall task due to interference from other targets cued by re-paired contexts. This pattern of interference in cued recall on the surface seems surprising, as none has been found in recognition studies. On the other hand, investigations of important memory phenomena that are present in recall but often absent from recognition, such as list-strength effect (Ratcliff, Clark, & Shiffrin, 1990; Verde, 2009), list-length effect (Dennis, Lee, & Kinnell, 2008), or retrieval-induced forgetting (Verde & Perfect, 2011), clearly suggest that interference effects play a more prominent role in tests of recall than recognition. This picture is, however, further clouded by the fact that all signatures of interference in the re-paired context condition were absent in Experiment 2. The only difference between these two experiments was the addition of a preexposure phase in the procedure and the inclusion of items from the prefamiliarized condition in the cued-recall test. Assuming that these differences between our experiments are not caused by a sampling error, it seems more likely that the change in the list composition could have been responsible for these divergent results. In Experiment 1 two thirds of contexts used in the cued-recall task were associated with one of the targets whereas in Experiment 2 only half of contexts were associated with any targets. Despite the fact that we did not ask our participants to retrieve targets associated with contexts and only required retrieval of targets associated with cues, it is possible that participants in Experiment 1 actively tried to gather any possible information during cued recall. By contrast, they could have been less motivated toward such active retrieval in Experiment 2 with fewer contexts associated with targets. Independently of whether this is the actual reason for our results, which is very speculative at best, it remains interesting that presenting re-paired context at retrieval can at least sometimes result in interference and reduced performance. The question whether such interference is automatic or requires participants to actively engage in an extended memory search should be the topic of further studies.

In conclusion, the results of the present study underscore the fact that metacognitive processing is sensitive to environmental context in which it takes place. The two context effects that have been previously described in studies on basic memory processes, the context familiarity effect and the context reinstatement effect, were revealed to affect FOK judgments in the present study. These findings demonstrate not only the need to broaden the investigations into the basis of metacognitive processing but also the usefulness of merging the metacognitive and memory approach to studying the process of remembering unfolding in various environmental contexts.

## Figures and Tables

**Table 1 tbl1:** Proportions of Correctly Recalled Targets in Cued-Recall Phase, Proportions of Intrusions in Cued Recall, Mean of FOK Judgments for Unrecalled Items, Mean Latencies to Respond Blank in Cued Recall (in Seconds), Mean Gamma Correlations Between FOK Judgments and Subsequent Recognition of Unrecalled Items, and Mean Hit Rates in a 6AFC Recognition for Unrecalled Items Presented as a Function of a Context Condition in Experiments 1 and 2

Experiment and condition	Reinstated context	Re-paired context	Novel context	Prefamiliarized context
Experiment 1				
Correct cued recall	.18 (.02)	.02 (.01)	.09 (.01)	—
Cued-recall intrusions	.20 (.03)	.31 (.03)	.18 (.03)	—
FOK judgments	38.7 (2.3)	35.4 (2.2)	30.5 (2.1)	—
*Blank* latencies	6.2 (.3)	6.0 (.4)	5.5 (.04)	—
FOK resolution	.13 (.08)	.00 (.07)	.04 (.08)	—
Recognition	.30 (.02)	.41 (.03)	.35 (.03)	—
Experiment 2				
Correct cued recall	.19 (.02)	.16 (.03)	.14 (.02)	.16 (.02)
Cued-recall intrusions	.10 (.02)	.10 (.02)	.09 (.02)	.09 (.02)
FOK judgments	43.6 (2.2)	40.1 (2.1)	36.3 (2.2)	45.0 (2.1)
*Blank* latencies	6.0 (.3)	5.8 (.03)	5.3 (.3)	5.9 (.3)
FOK resolution	.19 (.08)	.23 (.06)	.13 (.07)	.18 (.07)
Recognition	.44 (.03)	.42 (.03)	.39 (.03)	.40 (.03)
*Note.* Standard errors are given in parentheses. FOK = feeling of knowing; 6AFC = six-alternative forced-choice test.				

## Footnotes

1 Two participants were eliminated from this analysis due to missing cells.

2 Nine participants were eliminated from this analysis due to missing cells.

3 A reader may wonder why FOK judgments for the reinstated context condition were higher than in the re-paired context condition—a context reinstatement effect—while a similar comparison with the prefamiliarized context condition suggests a numerical difference in the opposite direction (see Table 1). It is crucial to stress here that contexts in these conditions were not equated in terms of familiarity. Contexts included at study, and then used in the reinstated and re-paired context conditions, were presented in the same way which was incidental to the focal task of memorizing pairs of words. Given the same format of presentation of these contexts in these two conditions, they are equated in terms of familiarity, facilitating the examination of the role of encoding-retrieval context match. By contrast, contexts subsequently used in the prefamiliarized context condition had been presented in a very different way, as focal elements in the deep-orienting task that is commonly used to produce strong memory traces. One could thus plausibly argue that such prefamiliarized contexts should be much more familiar than incidentally encoded contexts used in the reinstated context condition. In any case, the very different ways in which the incidental and the prefamiliarized contexts were encountered means that meaningful comparisons cannot be made in the way that comparisons between reinstated and re-paired contexts can.
